# The Influence of Starch Sweeteners on Functional Properties of Cellulose Fat Mimetics: Rheological and Textural Aspects

**DOI:** 10.3390/polym15142982

**Published:** 2023-07-08

**Authors:** Ivana Nikolić, Jovana Petrović, Biljana Pajin, Ivana Lončarević, Drago Šubarić, Đurđica Ačkar, Borislav Miličević, Zita Šereš, Ljubica Dokić, Dragana Šoronja-Simović, Antun Jozinović

**Affiliations:** 1 Faculty of Technology Novi Sad, University of Novi Sad, Bulevar Cara Lazara 1, 21000 Novi Sad, Serbia; ivananikolic@uns.ac.rs (I.N.); biljana.pajin@uns.ac.rs (B.P.); ivana.loncarevic@uns.ac.rs (I.L.); zita.seres@uns.ac.rs (Z.Š.); ldokic@uns.ac.rs (L.D.); dragana.soronja@uns.ac.rs (D.Š.-S.); 2 Faculty of Food Technology Osijek, Josip Juraj Strossmayer University of Osijek, Franje Kuhača 18, 31000 Osijek, Croatia; dsubaric@ptfos.hr (D.Š.); dackar@ptfos.hr (Đ.A.); ajozinovic@ptfos.hr (A.J.); 3 Faculty of Tourism and Rural Development Požega, Josip Juraj Strossmayer University of Osijek, Vukovarska 17, 34000 Požega, Croatia; bmilicevic@ftrr.hr

**Keywords:** microcrystalline cellulose, fat mimetics, starch sweetener, crystalline fructose, high-fructose corn syrup, rheology, texture

## Abstract

Starch sweeteners are commonly used in many confectionery food products. Usually, considering the trend of producing low-energy and low-fat products, these products include fat mimetics. The aim of this study was to investigate the influence of fructose sweeteners on the development of functional properties of MCG fat mimetic, such as rheological and textural behavior. Fat mimetics made from Microcrystalline cellulose gel (MCG) consist of colloidal microcrystalline cellulose (MCC) and sodium carboxymethyl cellulose (NaCMC) and were observed in five different concentrations (1, 3, 5, 7, and 10%). The amount of starch sweetener in the mixture with the fat mimetics was 20%. The effect of pure crystalline fructose and a mixture of crystalline-fructose and high-fructose corn syrup in a ratio of 1:1 was analyzed. Rheological parameters significantly decreased with the application of starch sweeteners. By adding a mixture of starch sweeteners, this decrease was further increased by 10%. At higher gel concentrations of 5, 7, and 10%, the dominance of the elastic modulus G′ was preserved. Texture parameters such as firmness, consistency, cohesiveness, and viscosity index were reduced accordingly. The presence of starch sweeteners significantly disrupted the networking of the three-dimensional structure of the MCG gel and the proper hydration process during the formation of fat mimetics.

## 1. Introduction

The development of functional food products focuses on a variety of low–energy food products, especially on products with reduced fat content, which is usually the most concentrated source of energy. Reducing the fat content and energy value of food products normally involves the application of fat mimetics. The “ideal” fat mimetic should have all of the functional characteristics of lipids, as well as a significantly lower energy value, preferably 0 kcal/g [1,2,3,4]. Fat mimetics can be based on proteins and carbohydrates that have been chemically or physically modified with the aim of performing the role of fat in food products. Cellulose-based fat mimetics are a group of carbohydrate fat mimetics with specific technological properties, such as water holding capacity (WHC), fat binding capacity, viscosity, gel forming ability, chelating ability, influence on product texture, etc. [5,6]. Microcrystalline cellulose (MCC) is one of the most common fat mimetics in food products, such as confectionery and bakery products, frozen desserts, salad dressings, fillings, cheeses, and spreads, due to its ability to provide the appropriate mouth feel, consistency, firmness, and structure [7,8,9]. The declaration code for microcrystalline cellulose in food products is “microcrystalline cellulose” or “cellulose gel”, and it is a GRAS substance (Generally Recognized As Safe) [10].

Sweeteners are also common components of food products, with sweet taste provided by special functional groups such as -OH, NH, and NO_2_. The ideal sweetener should be colorless, sweeter than sucrose and should leave a pleasant taste in the mouth. Furthermore, the ideal sweetener should be water soluble, stable in both acidic and basic environments and resistant to temperature changes. Also, the sweetener should be non-toxic, compatible with a wide range of products and other sweeteners, have a long shelf life, and be normally metabolized or excreted while remaining unchanged [11,12]. 

Many sweeteners are produced from various starch sources. Starch sweeteners include two groups of starch derivatives. The first group is starch hydrolysates, obtained only by the hydrolytic transformation of starch molecules, such as maltodextrins (DE < 20), starch syrups (DE = 20–75), dextrose hydrolysates (DE = 75–99.5), and crystalline D–glucose (DE > 99.5). The second group is sweeteners obtained via the further chemical transformation of starch hydrolysates from the first group, such as iso-sugars (high-fructose syrups), crystalline fructose, sugar alcohols, etc. [13,14,15,16]. Fructose-based starch sweeteners are sweeter than table sucrose, and thus are widely applicable. The most important functional properties of high-fructose corn syrups (HFCS) are taste enhancement, viscosity, water retention, fermentability, and the prevention of color development. HFCS are used to control crystallization, modify freezing point, increase osmotic pressure, and decrease water activity [17,18,19]. 

What differentiates crystalline fructose from sucrose, dextrose, corn syrups, and other sweeteners used in the food industry is its physical and physiological properties. Fructose manifests synergy with other sweeteners, and thus the relative sweetness of a mixture of fructose with sucrose, aspartame, saccharin, or sucralose is higher than the sweetness of the individual components of the mixture. The most important colligative properties are osmotic pressure, water activity, and freezing point reduction. Fructose has a higher osmotic pressure and lower water activity than sucrose, dextrose, and higher saccharides, and thus a greater microbiological stability. The lowering of the freezing point is more pronounced than with sucrose. Fructose absorbs moisture faster (hygroscopicity) and releases it more slowly into the environment (humectant) than sucrose, dextrose, and other sweeteners. Due to specific properties such as sweetness, low energy value, and solid aggregate state, fructose is used in various branches of the food industry [17,18,20,21].

Starch sweeteners are commonly used sweeteners in confectionery products. Given the trend of producing low-fat confectionery food products, and the necessary application of different fat mimetics during production, the main goal of this work was to observe the influence of starch sweeteners on development and functional properties of cellulose-based fat mimetics. Functional properties of fat mimetics include rheological and textural behavior because these systems are predominantly present in food products and play the role of a continuous phase.

## 2. Materials and Methods

### 2.1. Materials

The material used in the experiment was fat mimetic Vivapur MCG 611F produced by J. Rettenmaier & Sönhe GMBH + CO, Rosenberg, Germany. The chemical composition of Vivapur MCG 611F includes microcrystalline cellulose (MCC = 81.2–88.7%) and sodium carboxymethylcellulose (NaCMC = 11.3–18.8%). 

The starch sweeteners used in the experiment were crystalline-fructose and high-fructose corn syrup F42 (42% of fructose), both produced by Jabuka Starch Industry from Pančevo, Serbia. 

Distilled water was the other material used in the experiment. 

### 2.2. Preparation of Fat Mimetics

The fat mimetic powder was dispersed in distilled water under high shear of 6500 min^−1^ for 4 min using a homogenizer with the dispersing tool S25N–18G (Ultraturax T–25, IKA, Werke GmbH & Co., Staufen, Germany). Five different concentrations of colloidal microcrystalline cellulose were prepared: 1%, 3%, 5%, 7%, and 10%. The chosen concentrations should provide gel forms of MCG fat mimetics because the gel structure of fat mimetics can imitate the functional properties of fat [22,23]. The dispersed systems were stored at 4 °C for 24 h in order to form a gel structure.

### 2.3. Preparation of Mixture of Fat Mimetics and Starch Sweeteners

The effect of pure crystalline fructose as a starch sweetener was investigated, as well as the mixture of crystalline-fructose and high-fructose corn syrup in a 1:1 ratio. The combination of crystalline-fructose and high-fructose corn syrup is usually applied in order to achieve the adequate texture and sweetness of confectionery products that is not provided by pure crystalline fructose. The proportion of starch sweetener in the mixture with fat mimetics was 20% on fat mimetic mass. 

### 2.4. Rheological Determination

The rheological properties of the observed systems were defined using flow characteristics, dynamic oscillatory measurements, and creep and recovery analysis. All measurements were performed using the rotational viscometer HAAKE RheoStress RS600 (Thermo Electron Corporation, Karlsruhe, Germany) with a plate–plate sensor PP60 Ti (plate diameter was 60 mm and the gap was 1 mm) [24]. 

Flow properties were determined using the hysteresis loop method as dependence of shear stress (τ) over shear rate (γ). For three minutes, the samples were exposed to the shear rate increased from 0 to 100 s^−1^, followed by three minutes of constant shear rate at 100 s^−1^, and finally the sheer rate was decreased to 0 s^−1^ for 3 min. All measurements were carried out at 25 ± 0.1 °C [25]. 

Dynamic oscillatory measurements determined the elastic modulus (G′) and viscous modulus (G″) in the range of linear viscoelastic regime (LVE), where there is no destruction of the system and the values of applied shear stress depend on the strength of the system structure. The moduli were observed during the frequency increase from 1 to 10 Hz and at constant shear stress of 5 Pa in the linear viscoelastic regime. The results were expressed as the values tan δ = G″/G′ [26].

The creep and recovery test was used to determine the viscoelastic response of the samples under constant stress and after removing the stress, according to the compliance (J) of the samples. The creep and recovery tests were performed in the LVE regime, in which the deformation amplitude was proportional to the applied stress amplitude. The sample was exposed to constant stress (σ = 5 Pa) during creep time of 150 s. The recovery time after removing the stress was 450 s. The obtained data were analyzed using Burger’s model, which is presented in Equation (1) for the creep phase:

(1) $$J\left(t\right)=J_{0}+J_{1}\cdot \left[1-exp\left(-t/\lambda \right)\right]+t/\eta_{0}$$

and in Equation (2) for the recovery phase:

(2) $$J\left(t\right)=J_{max}-J_{0}-J_{1}\cdot \left[1-exp\left(-t/\lambda \right)\right]$$

where the value J_0_ is instantaneous compliance, J_1_ is retarded (viscoelastic) compliance, J_max_ is maximum compliance, λ is mean retardation time, and η_0_ is Newtonian viscosity [27,28,29,30,31].

### 2.5. Textural Determination

The textural properties of viscous liquid or semi-solid gel-like systems are commonly determined using methods of reverse or direct extrusion that define firmness and consistency [32,33]. Textural characteristics were determined using the Texture analyzer TA.HD Plus, Stable Micro Systems, Surrey, UK. The manufacturer’s specified method of Comparison of the consistencies by back extrusion was applied using the Back extrusion cell (A/BE) accessory, which is comprised of a base for positioning the sample container, sample containers with an internal diameter of 50 mm, a compression disk with a diameter of 35 mm and a 150 mm long disk holder. The method was performed with the following parameters: speed during the analysis was 1 mm/s, distance was 30 mm, contact force was 5 g, and the measuring cell was 5 kg.

The maximum realized force during disc penetration at a distance of 30 mm was used to define the firmness of the sample. The size of the area that the resulting curve built with the abscissa of the graph indicated the consistency of the gel. 

The negative part of the curve was obtained when returning the measuring equipment through the sample and described the resistance to the flow that the sample exhibited. The negative maximum of the curve was an indicator of its cohesiveness, and the negative area covered by the negative part of the curve and the abscissa represented the viscosity index. The textural parameters were calculated from the recorded graphs using Texture Exponent software, version 6.1.27.0, Stable Micro Systems, Surrey, UK [34].

### 2.6. Statistical Analysis

The measurements were performed three times for all determined rheological parameters and five times for the textural parameters. The obtained results were statistically analyzed by the ANOVA statistical method. The mean values were compared via a one-factor analysis of variance with Duncan’s post–hoc test at 5% level of significance, using software Statistica 13.3 (TIBCO Software Inc., Paolo Alto, CA, USA, 2016). 

Also, the linear relationship between individual variables was determined and expressed using the Pearson correlation coefficient, r, via a statistical method of linear correlation [35].

## 3. Results and Discussion

### 3.1. Rheological Properties of MCG Fat Mimetics

**Figure 1:**
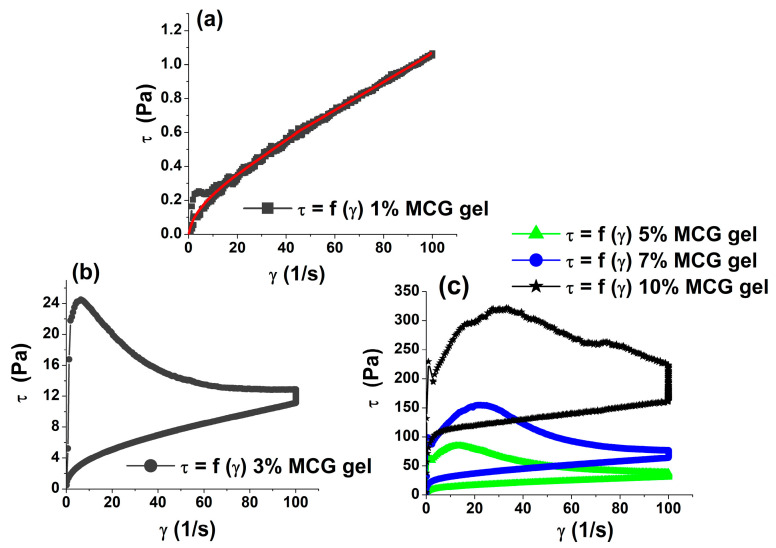
Flow curves of MCG fat mimetics with fiber concentrations of (**a**) 1%; (**b**) 3%; and (**c**) 5, 7 and 10%.

Figure 1 shows flow curves for MCG fat mimetics with fiber concentrations of 1, 3, 5, 7 and 10%; the gel of fat mimetics was formed at a fiber concentration higher than 1%.

The fibers of colloidal microcrystalline cellulose in an aqueous environment have the properties of hydrocolloids. Colloidal microcrystalline cellulose is water-insoluble and forms molecular dispersions with properties similar to hydrosoluble gums. Commercial colloidal microcrystalline cellulose is usually a mixture of MCC (microcrystal cellulose) and Na–CMC (sodium carboxymetil cellulose, cellulose gum), wherein the amount of cellulose gum ranges from 8.5 to 15% of the total amount of colloidal MCC [6]. When dispersed in water using sufficient shear, the microcrystalline cellulose (MCC) particles form a microscopic three–dimensional network of crystals. The coprocessed soluble hydrocolloids (Na–CMC) facilitate the formation of this network by acting as water swelling capillaries between the crystals, forcing them to open during hydration. Hydroxyl groups of macromolecules attract water molecules that form a water cylinder around the cellulose chains. This network is then stabilized by hydrogen bonding between the polar groups on the surface of the cellulose. The soluble hydrocolloids also function to consolidate the network via hydrogen bonding to the microcrystalline cellulose. This network imparts a unique rheology and structures water in a specific manner. The forces holding the network together are shear-sensitive and break down readily. When the shear is removed, the three-dimensional network quickly reforms giving microcrystalline cellulose dispersions marked thixotropic properties crystals is the key to the unique functionality of colloidal microcrystalline cellulose in f. This thixotropic network of insoluble crystals is the key to the unique functionality of colloidal microcrystalline cellulose in fat replacement and is stable over a wide range of pH values and temperatures [7,10,23,36,37].

At the lowest concentration of MCG fibers (1%), a pseudoplastic type of flow occurs, that is, shear stress changes are greater at lower shear rates than at higher shear rates. A pseudoplastic type of flow is characteristic of systems with solvated asymmetric colloidal particles, solutions of branched or linear macromolecules, etc. [26]. These systems do not cause yield stress, which is also confirmed by 1% MCG gel. 

The characterization of the rheology of colloidal MCC reveals that the formed gel possesses gel-like properties with a high degree of thixotropy [38]. As the gel concentration increases, so do the thixotropic characteristics of the networked gel structures of MCG fat mimetics. The degree of networking, which corresponds to the strength of the MCG gels [39], increases with the increase in concentration. This is indicated by the increase in the value of the rheological parameters. The rheological parameters of flow curves are yield stress τ_0_ (Pa) and the area of the thixotropic loop A_0_ (Pa/s), as presented in Table 1.

The viscoelastic nature of the above-mentioned gel systems is described by parameter tan δ (Table 1). It is defined as the ratio of G″ and G′ and measures the relative magnitude of viscous and elastic components of the system. The lower values of the tan δ indicate the more elastic nature of the system [31,39]. Rheological dynamic oscillatory measurements imply the dominance of elastic bonds in the structure of all MCG fat mimetics. Thus, the ratio of viscous, G″, and elastic, G′, modulus is less than 1. Colloidal MCC systems exhibit viscoelastic properties with a preponderance of the elastic over the viscous components under small-amplitude oscillatory shear, congruent with their microstructure [38]. Also, as the MCG fiber concentration increases, so does the influence of the elastic component, while the values of the tan δ parameter are decreased (Table 1). However, with the increase in the volume fraction of the particles, or increase in the solid gel phase, there is a pronounced increase in the modulus of elasticity and its dominance [40]. A higher number of fibers in the gel system causes intense intermolecular interactions, contributes to networking, increases the proportion of elastic components in the system, and indicates the formation of stable networked gel structures [41]. Given what has been said, these fat mimetics with observed concentrations from 1 to 10% are viscoelastic systems with dominant linkages of an elastic nature, with more or less pronounced stiffness of the system but a similar behavior.

### 3.2. Rheological Properties of Mixtures of MCG Fat Mimetics and Starch Sweeteners

#### 3.2.1. Flow Properties of Mixtures of MCG Fat Mimetics and Starch Sweeteners

**Figure 2:**
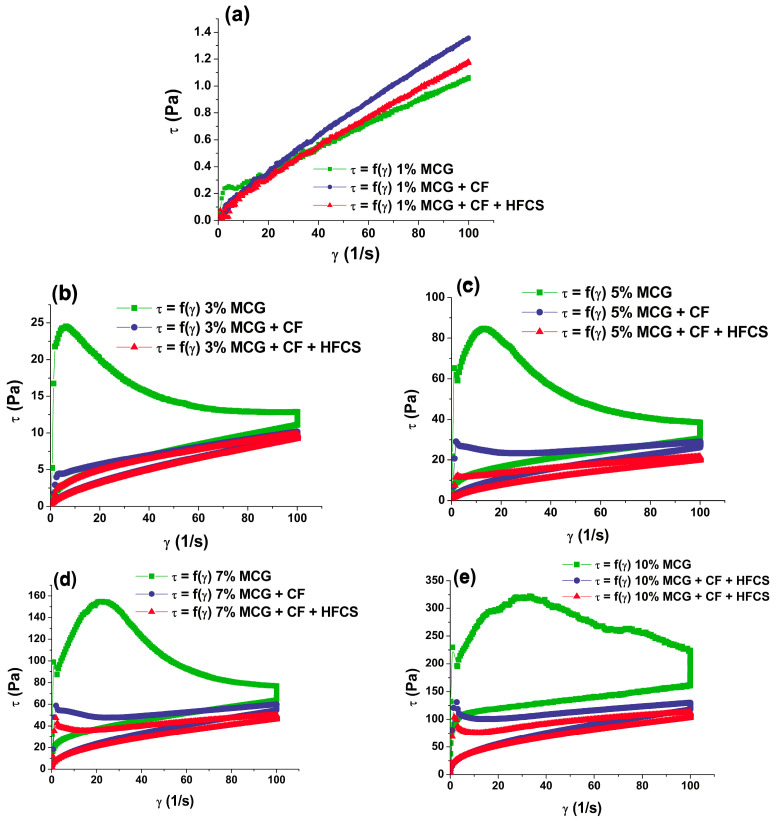
Flow curves of MCG fat mimetics with starch sweeteners. MCG gel concentrations are (**a**) 1%; (**b**) 3%; (**c**) 5%; (**d**) 7%; and (**e**) 10%.

Figure 2 shows the flow curves of mixtures of MCG fat mimetics and starch sweeteners. The starch sweeteners used were pure crystalline fructose and a mixture of high-fructose syrup and crystalline fructose. The pseudoplastic type of flow is characteristic of all curves. 

By observing the flow curve for MCG fat mimetics with a concentration of 1%, it can be noticed that the addition of sweeteners had little influence on the viscosity of mixtures, compared to flow curve of pure MCG fat mimetics (Figure 2a). The concentration of MCG macromolecules in the systems was low, whereby a weak gel structure with a large proportion of water was formed, and thus the flow properties of the system could be determined. The flow properties of the observed mixtures were not significantly affected by the small sweetener molecules. 

**Figure 3:**
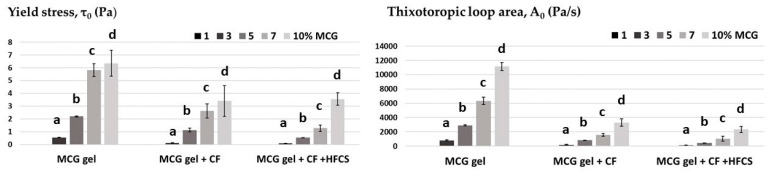
The changes in the flow parameters of mixtures of starch sweeteners and MCG fat mimetics compared to pure MCG gel. Within each observed fat mimetic, bar’s values with the same letter (a–d) are not significantly different (*p* > 0.05) according to Duncan’s test.

As the concentration of the MCG macromolecules increased (3, 5, 7, and 10%), the availability of free water in the system decreased; thus, stronger gel structures were formed (Figure 2b–e). The mixtures of starch sweeteners and MCG fat mimetics with fibers concentrations above 1% flowed with thixotropic properties, as well as pure MCG fat mimetics, indicating that gel structures were formed. However, the values of the flow curve parameters significantly changed after the starch sweeteners had been implemented into the fat mimetic structure. The applied sweeteners are very soluble in water, where they break down into fructose and dextrose molecules, i.e., the constituent monomers [17]. Their solubility significantly reduces the strength of the gel structure by disrupting the water cylinder of macromolecular chains and reduces the degree of networking of the gel. This is reflected in a statistically (*p* < 0.05) significant decrease in the values of yield stress, and areas of thixotropic loops of mixtures of MCG gel and starch sweeteners in relation to the values of the rheological parameters of pure MCG gels (Figure 3). A number of key factors may interfere with the proper dispersion of colloidal microcrystalline cellulose. Adequate shear must be used, i.e., the shear regime of the process must match the requirements of the grade of microcrystalline cellulose selected. Hard water/electrolytes can also inhibit the dispersion of colloidal microcrystalline cellulose, so dispersion in water containing low levels of salt is recommended. When acidifying a dispersion of microcrystalline cellulose to a pH below 4.5, a protective colloid is necessary to prevent flocculation [7]. 

The changes in the shown rheological parameters of flow curves were more pronounced with the application of the mixture of crystalline-fructose and high-fructose syrup as a sweetener, than with the use of pure crystalline fructose. The yield stress was decreased by 46.34–77.84% using just crystalline fructose, while the application of starch sweetener mixtures caused a reduction of yield stress by 23.24–84.25%. The reduction in the thixotropic loop area was greater and more pronounced with the implementation of starch sweetener mixtures (by 79.05 to 85.08%) than with the use of crystalline fructose (by 70.39–78.93%). Crystalline-fructose and high-fructose syrups differ both in their aggregate state and dry matter content. By using crystalline fructose, which contains up to 99.5% of dry matter, more dry matter was introduced into the analyzed gel system than when liquid high-fructose syrup with up to 71% of dry matter was used [42,43]. Therefore, the consistency of the mixture of MCG gel and crystalline fructose was slightly firmer than the consistency of the mixture with high-fructose syrup.

#### 3.2.2. Viscoelastic Properties of Mixtures of MCG Fat Mimetics and Starch Sweeteners

**Figure 4:**
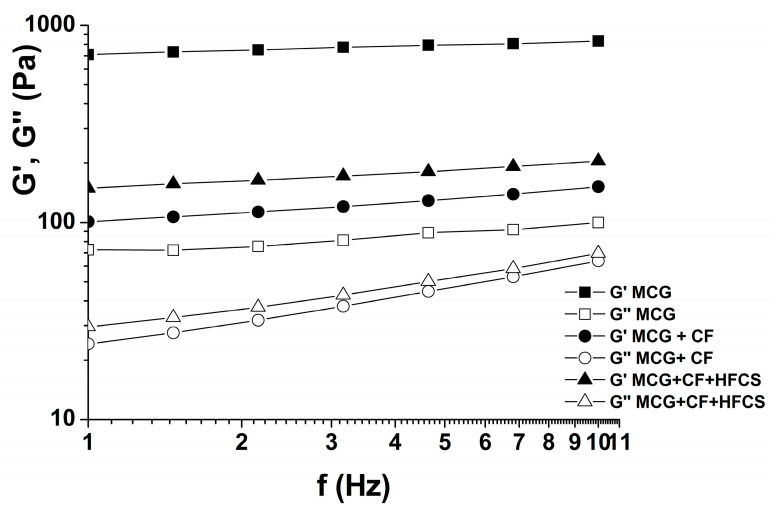
The changes in elastic G′ and viscous G″ moduli of gel systems with 10% MCG gel.

Table 2 presents the ratios of viscous, G″, and elastic, G′, moduli, i.e., the values of tan δ for the observed gel systems. The elastic modulus in all systems was higher than the viscous modulus (Figure 4).

All values of tan δ were less than 1 and had a statistically significant (*p* < 0.05) increase when the starch sweeteners were used. Thus, the mixtures of MCG gel and starch sweetener kept their viscoelastic properties, but they were reduced when compared to pure MCG gels. Accordingly, the utilization of starch sweeteners did not completely disrupt the gel structure of the system in comparison to the control MCG fat mimetics. The elastic bonds of the gel structure were not all broken by the application of starch sweeteners, thus there was no complete liquefaction of the system. Certainly, a higher fiber concentration in the MCG gel contributed to the elasticity and thus to the strength of fat mimetic structure, which was confirmed by the reduced values of tan δ. However, the reducing influence of the mixed starch sweetener on the elastic behavior of MCG gel was more pronounced than with crystalline fructose, which resulted in the higher increase of tan δ and its values, which approached 1 (Table 2).

#### 3.2.3. Creep and Recovery Curves of Mixtures of MCG Fat Mimetics and Starch Sweeteners

Creep and recovery curves demonstrate the ability of the system to resist constant stress and the possibility of recovering after the applied stress. Accordingly, the viscoelastic behavior of the system influenced by the application of starch sweeteners was also analyzed using this rheological method. The obtained creep and recovery curves display MCG fat mimetics with concentrations of 3% and higher as viscoelastic systems, which have a certain ability to resist the constant applied stress and the ability to recover after the stress. These properties of the MCG gel were caused by the presence, number, and strength of elastic bonds in the gel structure [44], so they became more pronounced with the increase in the gel concentration. The low MCG concentration of 1% did not have creep and recovery abilities. 

The implementation of starch sweeteners into the MCG gel structure of all the examined concentrations reduced the ability of the system to resist constant stress and decreased the ability of recovery.

**Figure 5:**
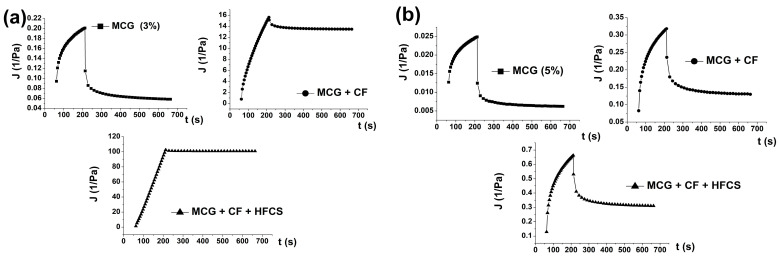
The changes of creep and recovery curves with the application of starch sweeteners in MCG gel structure with (**a**) 3% of fibers and (**b**) 5% of fiber concentration.

This phenomenon was most pronounced in MCG gels of low concentration (3%), (Figure 5a). Due to the disruption of elastic bonds after the application of sweeteners, the number of viscous deformations in the system after the recovery phase was significantly higher. The creep curve of the system consisting of 3% MCG gel and mixed starch sweeteners was very close to the theoretical curve of an ideal viscous body (Figure 5a) [26]. The obtained creep curves with high coefficients of determination, R^2^, corresponded to the equations of Burger’s model, which are shown in Table 3. This influence of sweeteners was confirmed by all parameters of Burger’s model, as shown in Table 3. The values of compliance, J_0_, J_1_, and J_max_, decreased with the increase in the concentration of pure MCG gel for the whole order of magnitude, while the value of Newtonian viscosity η_0_ increased, due to the strengthening of their consistency. When the starch sweeteners were used, these values changed in the opposite direction, which indicated a weakening of fat mimetic consistency.

### 3.3. Textural Properties of the Mixtures of MCG Fat Mimetics and Starch Sweeteners

The textural method, applied to determine the gel system via reverse extrusion, is numerically expressed and used to compare textural properties, such as firmness, consistency, cohesiveness, and viscosity index. Firmness and consistency are directly related to the required force to break the structure of the analyzed systems and indicate the strength of the structural connections. Thus, these measurements are a destructive type of measurement, applied beyond the breaking limits [32,33]. Cohesiveness and viscosity index refer to the connection of the constituents and the resistance of the system during the flow. Therefore, they are determined during the return phase of the measuring tool through the sample [45], and their values are negative. The obtained results for cohesiveness and index of viscosity were observed as absolute values. 

**Figure 6:**
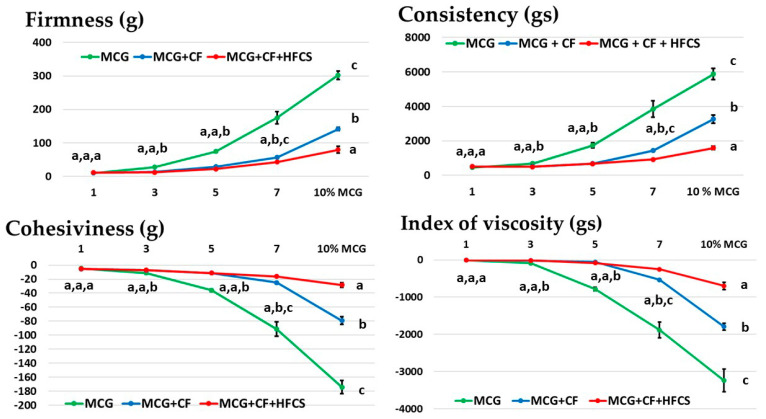
Changes in the textural parameters of mixtures of starch sweeteners and MCG fat mimetics. Presented points on diagrams are mean values of textural parameters ± standard deviations. Values with the same letter (a–c) are not significantly different (*p* > 0.05) according to Duncan’s test.

Figure 6 shows the changes in textural parameters when the gel concentration was changed compared to when the starch sweeteners were applied. The increase in the concentration of pure MCG gel contributed to the increase in the value of all examined textural parameters. With the increase in the amount of MCG macromolecules in the aqueous medium, the degree of networking was increased, and thus the strength (consistency) and cohesiveness of the gel structure of fat mimetics increased as well.

In the mixtures of MCG fat mimetics and starch sweeteners, the values of these parameters significantly decreased (*p* < 0.05), except in the systems with an MCG gel concentration of 1%, where the values of these parameters differed insignificantly (*p* > 0.05). This was the result of the weak and unstable structure of the 1% MCG gel, which was easily damaged by the use of starch sweeteners, thus resulting in the fat mimetic structure with liquid consistency and extremely low viscosity. MCG gels of higher concentrations retained a certain firmness and cohesiveness even after the application of starch sweeteners but with lower values of textural parameters than in pure MCG gels. In the gel systems with a mixture of starch sweeteners (CF + HFCS), this change was more noticeable, and thus there was a greater decrease in textural parameters. 

A linear correlation between the determined parameters was defined using the Pearson correlation coefficient (r). A linear correlation was observed between rheological viscoelastic characteristics, determined under conditions of small deformation, and the flow and textural properties, determined under conditions of large deformation. All statistically significant (*p* < 0.05) correlations were presented in Table 4.

High positive-correlation coefficients between the parameters of large deformation tests, such as yield stress, thixotropic loop area and textural characteristics, confirmed that the firmness and hard consistency of fat mimetics depends on gel cohesiveness and macromolecule networking. This resulted in an increase in yield stress and thixotropy during flow. A negative correlation between tan δ and the other observed parameters confirmed that the observed fat mimetics were viscoelastic systems, in which the contribution of elastic linkages conditioned the strength of the structure and the level of ability to recover.

Also, high correlation coefficients indicated cooperativity between textural and rheological measurements in defining the rheological behavior of the gel system, which was also mentioned by Lazaridou and Biliaderis [30] as well as Roopa and Bhattacharya [46]. 

## 4. Conclusions

Colloidal microcrystalline cellulose with MCG fiber concentrations higher than 1% formed stable gel structures in water. The obtained gels showed thixotropic flow characteristics and viscoelastic structures.

By introducing starch sweeteners into the MCG gel structure, the values of the observed rheological and textural parameters decreased when compared to the pure MCG gels. The dominance of the elastic component in the viscoelastic structure significantly decreased; thus, the creep and ability of the system to recover were reduced. This was the result of a partial disruption of the gel structure and a decrease in the degree of crosslinking of MCG fibers. Fructose molecules of starch sweeteners obstructed the interaction between the functional groups of cellulose and their networking ability. The influence of the mixture of sweeteners (crystalline-fructose and high-fructose corn syrup) on all the observed parameters was greater than the influence of pure crystalline fructose as sweeteners.

The abovementioned negative effects of starch sweeteners on the rheological and textural properties of cellulose fat mimetic gels, can be avoided by adding raw materials in the correct order during production. This can enable the proper hydration of the MCG gel before the addition of small-molecule components.

## Figures and Tables

**Table 1:** Rheological parameters of MCG fat mimetics.

MCG Fat Mimetics Concentration %	Yield Stress, τ_0_ (Pa) *	Thixotropic Loop Area, A_0_ (Pa/s) *	Tan δ *, G″/G′
1	0.033 ± 0.01 a	23.16 ± 4.42 a	0.656 ± 0.065 e
3	0.546 ± 0.05 b	784.00 ± 98.85 b	0.263 ± 0.010 d
5	2.195 ± 0.01 c	2909.00 ± 62.45 c	0.140 ± 0.008 c
7	5.521 ± 1.42 d	6037.33 ± 509.56 d	0.124 ± 0.003 b
10	7.203 ± 0.50 e	6717.33 ± 567.71 e	0.107 ±0.006 a

* Values are presented as mean value of 3 measurements ± standard deviation. a–e Values followed by the same letter within the same column are not significantly different (*p* > 0.05) according to Duncan’s test.

**Table 2:** The values of tan δ (*) for mixtures of starch sweeteners and fat mimetics.

Samples	MCG Fat Mimetic Concentration (%)				
	1	3	5	7	10
MCG gel	0.556 ± 0.065 a	0.263 ± 0.021 a	0.144 ± 0.008 a	0.124 ± 0.003 a	0.107 ± 0.006 a
MCG + CF	0.934 ± 0.011 b	0.672 ± 0.016 b	0.453 ± 0.006 b	0.320 ± 0.002 b	0.139 ± 0.006 b
MCG + CF + HFCS	0.961 ± 0.013 b	0.804 ± 0.018 c	0.585 ± 0.001 c	0.458 ± 0.009 c	0.299 ± 0.006 c

* Values are presented as mean value of 3 measurements ± standard deviation. a–c Values followed by the same letter within the same column are not significantly different (*p* > 0.05) according to Duncan’s test.

**Table 3:** The values of creep and recovery parameters obtained from Burger’s model.

Sample of Fat Mimetics	Creep Phase					
	J_0_ 10^−2^ (1/Pa)	J_1_ 10^−2^ (1/Pa)	η_0_ 10^2^ (Pas)	λ_1_ (s)	J_max_ 10^−2^ (1/Pa)	R^2^
3% MCG	9.25	6.45	10.88	92.34	19.55	0.7970
3% MCG + CF	82.02	32.25	0.12	92.44	1734	0.6955
3% MCG + CF + HFCS	130.1	58.22	0.22	92.31	9761	0.6936
5% MCG	1.26	0.81	87.28	92.33	2.43	0.9960
5% MCG + CF	8.06	10.96	6.41	92.39	33.21	0.9970
5% MCG + CF + HFCS	13.53	22.26	3.15	92.46	67.45	0.9949
7% MCG	0.44	0.30	235.35	92.33	0.90	0.9969
7% MCG + CF	3.44	3.59	20.16	92.36	10.90	0.9971
7% MCG + CF + HFCS	4.27	5.01	15.72	92.28	15.39	0.9970
10% MCG	0.15	0.11	613.71	92.42	0.35	0.9965
10% MCG + CF	1.12	0.96	73.45	92.31	2.89	0.9939
10% MCG + CF + HFCS	1.16	0.95	71.25	92.40	2.92	0.9918
	Recovery phase					
	J_0_ 10^−2^ (1/Pa)	J_1_ 10^−2^ (1/Pa)	η_0_ 10^2^ (Pas)	λ_1_ (s)	R^2^	
3% MCG	11.13	1.84	11.90	288.55	0.7210	
3% MCG + CF	17.34	5.17	0.43	288.55	0.6089	
3% MCG + CF + HFCS	974.6	31.69	0.69	288.45	0.6970	
5% MCG	1.15	0.94	17.62	288.45	0.8950	
5% MCG + CF	24.77	4.49	4.88	288.55	0.8081	
5% MCG + CF + HFCS	54.10	10.43	2.10	288.50	0.8930	
7% MCG	0.47	0.13	240.30	288.60	0.9700	
7% MCG + CF	7.52	1.27	18.42	288.55	0.9761	
7% MCG + CF + HFCS	11.41	2.12	12.92	288.55	0.9388	
10% MCG	0.21	0.34	414.60	288.45	0.9886	
10% MCG + CF	1.81	0.51	65.10	288.60	0.9166	
10% MCG + CF + HFCS	1.90	0.56	69.49	288.55	0.9006	

**Table 4:** Linear correlation between the rheological and textural parameters of the mixtures.

	Yield Stress	Thixotropic Area	Firmness	Consistency	Cohesiveness	Index of Viscosity	Tan δ	Newtonian Viscosity
Yield stress	1.0000	0.9157	0.9268	0.9224	0.8894	0.9183	−0.7798	0.8190
Thixotropic area		1.0000	0.9812	0.9610	0.9753	0.9577	−0.6576	0.9746
Firmness			1.0000	0.9920	0.9942	0.9836	−0.6835	0.9408
Consistency				1.0000	0.9887	0.9835	−0.6810	0.9071
Cohesiveness					1.0000	0.9804	−0.6290	0.9470
Index of viscosity						1.0000	−0.6875	0.9063
Tan δ							1.0000	−0.5233
Newtonian viscosity								1.0000

## Data Availability

Not applicable.
